# Improving health literacy of antibiotic use in people with cystic fibrosis (CF)—comparison of the readability of patient information leaflets (PILs) from the EU, USA and UK of 23 CF-related antibiotics used in the treatment of CF respiratory infections

**DOI:** 10.1093/jacamr/dlad129

**Published:** 2023-12-01

**Authors:** Ka Wah Kelly Tang, Beverley C Millar, John E Moore

**Affiliations:** School of Biomedical Sciences, Ulster University, Cromore Road, Coleraine BT52 1SA Northern Ireland, UK; School of Biomedical Sciences, Ulster University, Cromore Road, Coleraine BT52 1SA Northern Ireland, UK; Laboratory for Disinfection and Pathogen Elimination Studies, Northern Ireland Public Health Laboratory, Belfast City Hospital, Lisburn Road, Belfast BT9 7AD Northern Ireland, UK; Northern Ireland Regional Adult Cystic Fibrosis Centre, Level 8, Belfast City Hospital, Lisburn Road, Belfast BT9 7AB, Northern Ireland, UK; School of Biomedical Sciences, Ulster University, Cromore Road, Coleraine BT52 1SA Northern Ireland, UK; Laboratory for Disinfection and Pathogen Elimination Studies, Northern Ireland Public Health Laboratory, Belfast City Hospital, Lisburn Road, Belfast BT9 7AD Northern Ireland, UK; Northern Ireland Regional Adult Cystic Fibrosis Centre, Level 8, Belfast City Hospital, Lisburn Road, Belfast BT9 7AB, Northern Ireland, UK

## Abstract

**Background:**

Antibiotic adherence is poor amongst people with cystic fibrosis (CF). Low-quality patient information leaflets (PILs), which accompany prescription antibiotics, with poor readability may contribute to poor antibiotic adherence, with the potential for antimicrobial resistance (AMR) development. The aim of this study was to examine the readability of antibiotic PILs used to treat CF lung infections.

**Methods:**

CF-related antibiotics (*n* = 23; seven classes: aminoglycosides, β-lactams, fluoroquinolones, macrolides/lincosamides, oxazolidinones, tetracyclines, trimethoprim/sulfamethoxazole) were investigated. Readability of PILs (*n* = 141; 23 antibiotics) from the EU (*n* = 40), USA (*n* = 42) and UK (*n* = 59) was calculated.

**Results:**

Mean [± standard error of mean (SEM)] values for the Flesch Reading Ease (FRE) for EU, USA and UK were 50.0 ± 1.1, 56.2 ± 1.3 and 51.7 ± 1.1, respectively (FRE target ≥60). Mean (± SEM) values for the Flesch Kinkaid Grade Level (FKGL) for the EU, USA and UK were 9.0 ± 0.2, 7.5 ± 0.2 and 9.6 ± 0.2, respectively (FKGL target ≤8). US PILs were significantly shorter (*P* < 0.0001) in words (mean ± SEM = 1365 ± 52), than either UK or EU PILs, with fewer sentences (*P* < 0.0001), fewer words per sentence (*P* < 0.0001) and fewer syllables per word. The mean ( ± SEM) reading time of UK PILs (*n* = 59) was 12.7 ± 0.55 mins .

**Conclusions:**

Readability of antibiotic PILs is poor. Improving PIL readability may lead to improved health literacy, which may translate to increased antibiotic adherence and AMR avoidance. Authors preparing written materials for the lay/patient CF community are encouraged to employ readability calculators, so that final materials are within recommended readability reference parameters, to support the health (antibiotic) literacy of their readers.

## Introduction

Cystic fibrosis (CF) is an inherited disorder caused by mutations in the gene that codes for the CF transmembrane conductance regulator (CFTR) protein, which results in the malfunction of the CFTR protein.^1^ The dysfunction of the CFTR protein leads to the development and accumulation of thick mucus, particularly in the lungs, which greatly restricts mucociliary clearance. For a full description on the pathophysiology of CF, see the review by Shteinberg and collegaues.^2^ Such limited or absent CFTR functionality contributes to creating conditions for commensal, environmental and pathogenic bacteria, including *Staphylococcus aureus* and *Pseudomonas aeruginosa*, to colonize, survive and persist, potentially leading to chronic infection.^3^ As a result, people with CF (PwCF) are particularly vulnerable to lung infections, which are responsible for increased morbidity and mortality. Pulmonary disease continues to be the main cause of death for CF patients.^4^ Since PwCF are often associated with chronic infections, such infections often require continual treatment with antibiotics. Frequent exposure to a range of different antibiotics, in a variety of formulations, including oral, nebulized/dry powder inhalation and IV, are usually part of daily treatment for CF-related lung infections. Despite this, antibiotic treatment often fails to completely eliminate the pathogens involved and this promotes the emergence of antimicrobial resistance (AMR).^5^

Given the continuous and complex approaches to therapy within CF, particularly at home, as well as during inpatient stays in hospital, mainly for the administration of IV antibiotics, such therapies generate a large burden of treatment, which patients often struggle to fully adhere to.^6^ Several studies that have examined patient adherence have reported ranges from 35% to 75%, while respiratory medication adherence ranges from 31% to 79%.^7,8^

Other research has indicated that the lack of adherence could be associated with lower quality of life.^9^ An earlier study has shown that patient compliance with the recommended course of therapy could have a potential impact on understanding and improving treatment outcomes.^10^ A further study has shown that CF patients exhibit poor self-management of medications and take fewer antibiotics than advised.^11^ There are many reasons that could contribute to poor antibiotic compliance and adherence in PwCF. The most common reason stated in previous studies is forgetfulness.^12^ A more recent study has also found that a potential barrier consistently present involves social life and travels, where some CF patients are found unwilling to take or are unable to take certain more complicated antibiotics such as nebulizer treatments.^13^ It is also recognized that there is a high treatment burden in CF.^14^ The increasing time of daily treatments could also lead to poor compliance, and poor compliance with prescribed antibiotics, especially in CF patients, raises the risk of developing antibiotic resistance in those pathogens that chronically infect the CF lungs.

One aspect of the study of poor patient compliance with antibiotic therapy that has not been examined to date has been how well patient information leaflets (PILs) are written for patients, parents of CF children with the responsibility of administering medicine to their child outside hospital, as well as with carers. Another potential factor that may potentially affect patient antibiotic adherence is the impact of the readability of these antibiotic PILs. PILs are enclosed with prescription drugs by the dispensing pharmacist and these are crucial in providing key information about dose, administration, side effects and safety precautions. A previous study has indicated the importance of evaluating the readability of PILs attached to medication, as low-quality information provided could potentially lead to increased patient misuse and cause lower adherence to taking antibiotics correctly.^15^ The consequence of poor adherence to antibiotics could potentially lead to the development of AMR, due to the presence of suboptimal MICs of antibiotics, and thus lead to poor health outcomes. A recent *in vitro* study demonstrated the relationship between suboptimal concentrations of tobramycin and the development of tobramycin resistance in *P. aeruginosa*.^16^

Readability can be assessed through a range of quantitative readability parameters and formulae based on various text metrics such as word count, sentence count and syllables (see Table S1, available as Supplementary data at *JAC-AMR* Online). Some readability formulae commonly used in healthcare include the Flesch-Kincaid Grade Level (FKGL) and the Flesch Reading Ease (FRE) scores. For the FKGL, a target score of eighth grade or lower is desirable, whilst the target score for the FRE is ≥60.

For definition purposes, the FKGL eighth grade is based on the US educational system and relates to children 13–14 years old (equivalent to Year 9 in the UK educational system). For further comparisons of age and grade levels, see Table S2.

To date, there has not been any research conducted that has examined the readability of patient-facing materials, including PILs for CF-related antibiotics. If antibiotic patient information that accompanies those antibiotics is poor, then the patients may be less likely to understand how and why they should take their antibiotics, which may result in them not properly or consistently taking their antibiotics as required.

A good understanding of medication instruction is vital for an individual to adequately comprehend and follow the recommended intake and dose of medication, in a way to maximize health outcomes and an additional way to minimize potential contributing factors to AMR. The aim of this study was therefore to examine the readability (FRE, FKGL, Gunning Fog, SMOG scores; text metrics) of PILs of CF-related antibiotics (*n* = 23; seven antibiotic classes including aminoglycosides, β-lactams, fluoroquinolones, macrolides/lincosamides, oxazolidinones, tetracyclines and trimethoprim/sulfamethoxazole) used to treat respiratory infections, from three geographical regions (UK, EU, USA), in order to establish: (i) how readable these patient-facing antibiotic resources from each geographical region compared with readability reference standards; (ii) if there are differences in readability between the different classes of antibiotics examined; and (iii) if there are differences in readability between antibiotics that are administered via oral, IV and nebulized routes.

## Materials and methods

**Figure 1.**
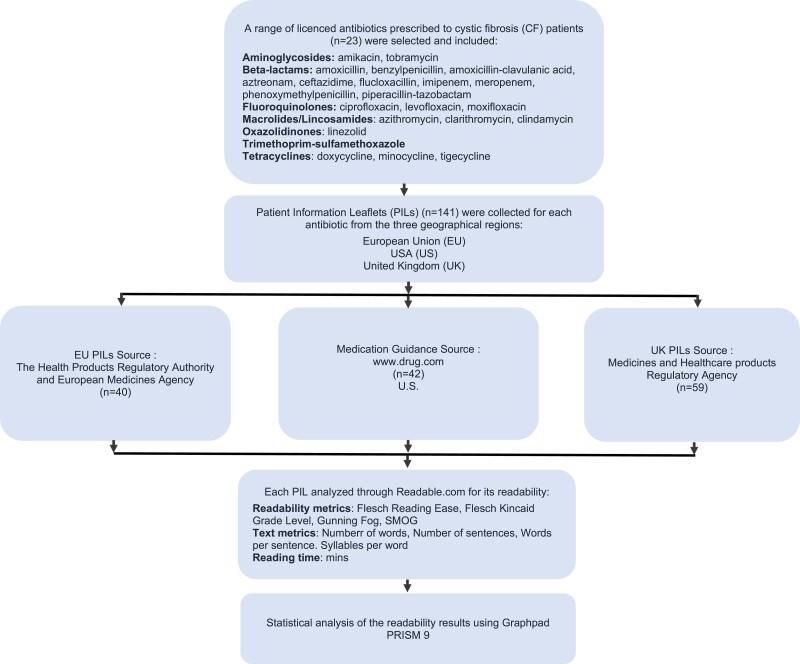
Flow diagram of methodological investigations undertaken in this study.

An overview of the methods employed is shown in Figure 1.

### Retrieval of CF-related antibiotic PILs

CF-related antibiotics (*n* = 23), which are used to treat CF-related bacterial respiratory infection, including non-tuberculous mycobacterial (NTM) infection, were selected for investigation.^17,18^ These included: aminoglycosides (amikacin, tobramycin), fluoroquinolones (ciprofloxacin, levofloxacin, moxifloxacin), β-lactams (amoxicillin, benzylpenicillin, amoxicillin/clavulanic acid, aztreonam, ceftazidime, flucloxacillin, imipenem, meropenem, phenoxymethylpenicillin, piperacillin/tazobactam), trimethoprim/sulfamethoxazole, macrolides/lincosamides (azithromycin, clarithromycin, clindamycin), oxazolidinones (linezolid) and tetracyclines (doxycycline, minocycline, tigecycline).

PILs (*n* = 141) aimed at patients and the general public were obtained from publicly and freely available web resources, during the period January 2023–March 2023. EU PILs were sourced from the Health Products Regulatory Authority (HPRA) (https://www.hpra.ie)/EMA (https://www.ema.europa.eu/en) websites. For the USA, patient information was collected from drugs.com (www.drugs.com), which is the most popular and up-to-date source of drug information, widely used by the general public to source any drug-related information. The UK PILs were sourced from the Medicines and Healthcare products Regulatory Agency (MHRA) website (https://www.gov.uk/government/organisations/medicines-and-healthcare-products-regulatory-agency). A total of 40 PILs were collected for the EU, 42 PILs for the USA and 59 were collected from the UK.

### Determination of readability scores and text metrics

Each PIL was examined using the online subscription-based software, *Readable* (www.readable.com), which was used in accordance with the website’s instructions. All PILs examined were written in English. The software was used to calculate four readability scores, including: (i) FRE; (ii) FKGL; (iii) Gunning fog index; and (iv) SMOG index, as detailed in Table S1. An additional four text metrics were also calculated, including word count, sentence count, words per sentence and syllables per word. These readability measures were chosen for examination as most readability studies frequently employ these. The reading time (mins) required to read each UK PIL was calculated. Finally, antibiotics were subdivided by (i) antibiotic class and (ii) drug formulation [oral/IV/nebulized (including dry powder inhalers)] and the readability examined.

Readable.com was selected as the preferred online calculator, as it has been previously used in several healthcare readability studies,^19,20^ as well as in a recent study that compared a variety of online readability calculators and concluded that *Readable* was the optimum calculator to use due to its accuracy, user experience and capacity to examine multiple readability parameters from clinical materials.^21^

### Statistical analyses

The readability data obtained underwent statistical analyses using GraphPad Prism version 9 (Boston, USA). To determine if the data followed a normal distribution, a normality test was performed on each set of data using the Shapiro–Wilk test. For datasets that were not normally distributed, the Kruskal–Wallis (non-parametric) test with Dunn’s adjusted *P* values was performed. A *P* value of <0.05 (5%) was considered as statistically significant.

### Ethics

This study did not involve human or animal subjects. All of the material used in this study was openly and freely available to the public and within the public domain.

## Results

### Comparison of readability scores and text metrics of CF antibiotic PILs from the EU, USA and UK

**Figure 2.**
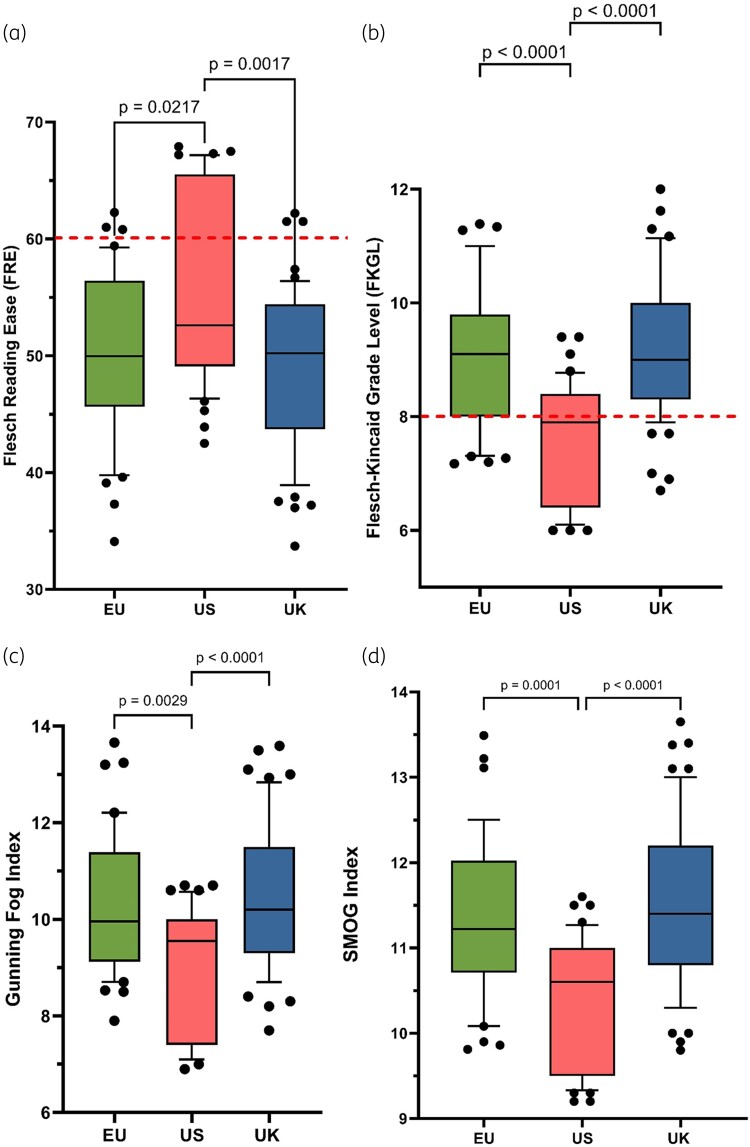
Box-and-whisker plots comparing readability scores calculated from CF-related antibiotics (*n* = 23; seven antibiotic classes), from the EU (*n* = 40), USA (*n* = 42) and UK (*n* = 59). (a) FRE; (b) FKGL; (c) Gunning fog score; and (d) SMOG score. Boxes represent IQRs (25^th^ to 75^th^ percentiles) and bars represent the medians. Whiskers represent the 10^th^ and 90^th^ percentiles and black dots represent outliers outside these percentile ranges. Statistical significance is shown, calculated using the Kruskal–Wallis (non-parametric) test with Dunn’s adjusted *P* values. A *P* value of <0.05 (5%) was considered as statistically significant. The dashed red lines represent the target readability score. For the FRE, this is ≥60. For the other scores, this is ≤8.

**Figure 3.**
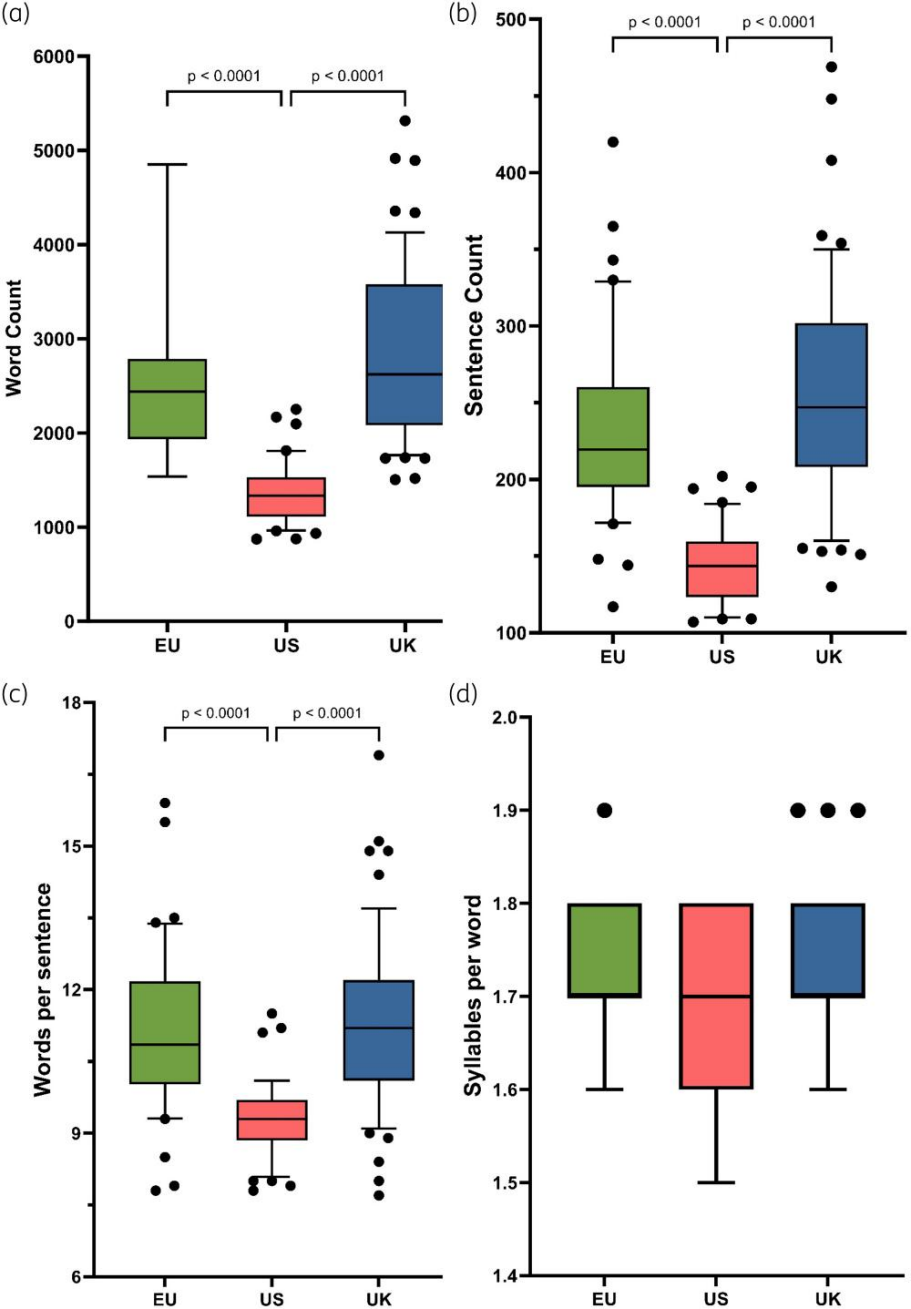
Box-and-whisker plots comparing text metric scores calculated from CF-related antibiotics (*n* = 23; seven antibiotic classes), from the EU (*n* = 40), USA (*n* = 42) and UK (*n* = 59). (a) Word count; (b) sentence count; (c) words per sentence; and (d) syllables per word. Boxes represent IQRs (25^th^ to 75^th^ percentiles) and bars represent the median. Whiskers represent the 10^th^ and 90^th^ percentiles and black dots represent outliers outside these percentile ranges. Statistical significance is shown for all comparisons, calculated using the Kruskal–Wallis (non-parametric) test with Dunn’s adjusted *P* values. A *P* value of <0.05 (5%) was considered as statistically significant.

**Figure 4.**
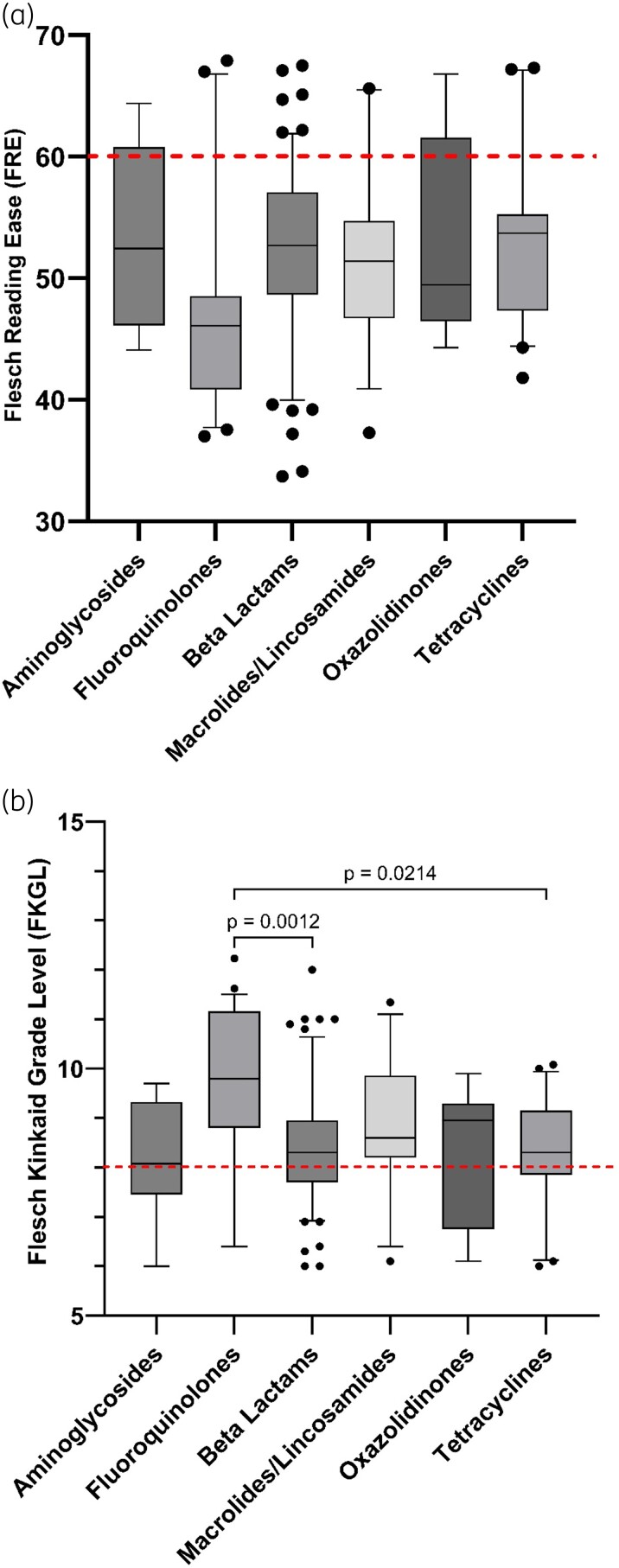
Box-and-whisker plots comparing the FRE score (a) and FKGL score (b) for six classes of CF-related antibiotics. Boxes represent the IQRs (25^th^ to 75^th^ percentiles) and bars represent the medians. The dashed red lines represent the target readability score (FRE ≥60; FKGR ≤ 8). Whiskers represent the 10^th^ and 90^th^ percentiles and black dots represent outliers outside these percentile ranges. Statistical significance is shown, calculated using the Kruskal–Wallis (non-parametric) test with Dunn’s adjusted *P* values. A *P* value of <0.05 (5%) was considered as statistically significant.

**Figure 5.**
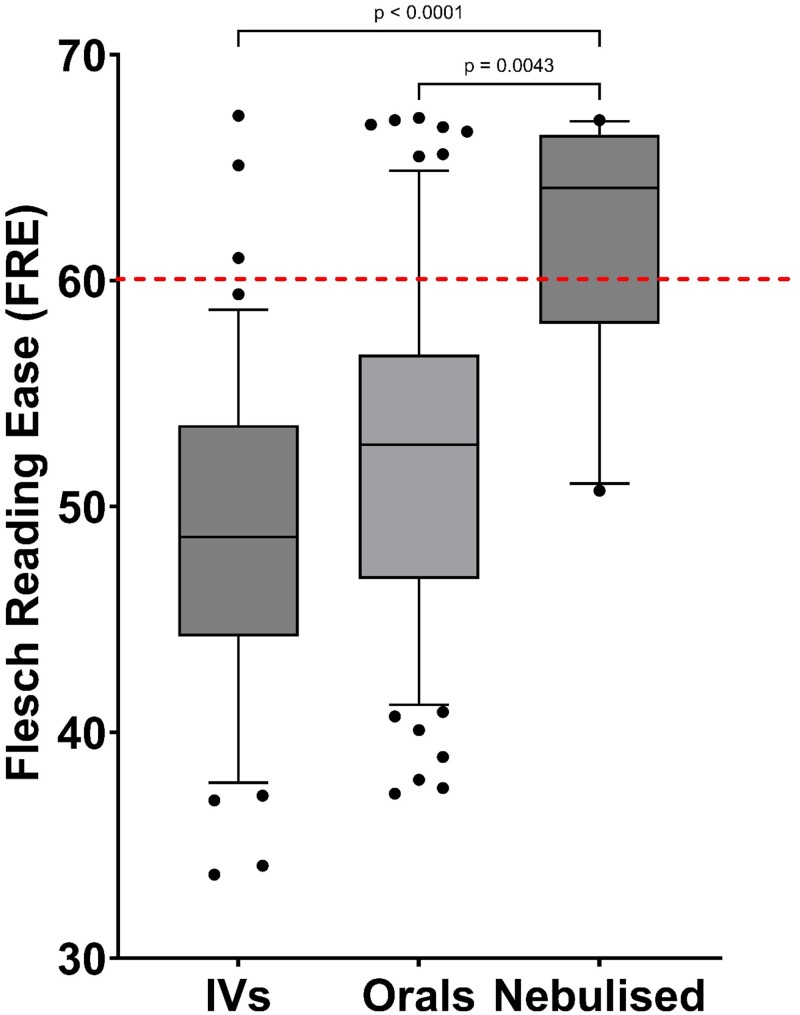
Box-and-whiskers plot comparing the FRE score for oral (*n* = 77), IV (*n* = 42) and nebulized (*n* = 10) CF-related antibiotics. Boxes represent the IQRs (25^th^ to 75^th^ percentiles) and bars represent the medians. Whiskers represent the 10^th^ and 90^th^ percentiles and black dots represent outliers outside these percentile ranges. The dashed red line represents the target readability score (FRE ≥60). Statistical significance is shown, calculated using the Kruskal–Wallis (non-parametric) test with Dunn’s adjusted *P* values. A *P* value of <0.05 (5%) was considered as statistically significant.

A total of 141 antibiotic PILs, relating to those antibiotics commonly prescribed for the treatment of respiratory infections in people with CF, were analysed, included PILs from the EU (*n* = 40), USA (*n* = 42) and UK (*n* = 59). All datasets were found to be not normally distributed, therefore for comparison, the Kruskal–Wallis test and Dunn’s multiple comparisons test were used to compare readability parameters between each geographical region. Readability scores for the FRE, the FKGL, the Gunning fog score and the SMOG score, for each geographical region, are shown in Figure 2(a–d). Text metrics, including word count, sentence count, words per sentence and syllables per word, for each geographical region, are shown in Figure 3(a–d). Additional readability analyses of PILs information, based antibiotic class (Figure 4a and b) and route of administration (oral versus IV versus nebulized) was determined (Figure 5).

Overall by combining the EU, US and UK data, the mean [±standard error of mean (SEM)], length of PILs was 2338 words (±85.7), comprising a mean (±SEM) sentence count of 215.2 ± 6.4, 10.7 ± 0.2 words per sentence and 1.7 ± 0.01 syllables per word. The mean (±SEM) reading time of UK PILs (*n* = 59) was 12.7 ± 0.55 mins.

By geographical region, the mean (±SEM) values for the FRE for the EU, USA and UK were 50.0 ± 1.1, 56.2 ± 1.3 and 51.7 ± 1.1, respectively (FRE target ≥60). Mean (±SEM) values for the FKGL for the EU, USA and UK were 9.0 ± 0.2, 7.5 ± 0.2 and 9.6 ± 0.2, respectively (FKGL target ≤8). US PILs were significantly shorter (*P* < 0.0001) in words (mean ± SEM = 1365 ± 52) than either the UK (2858 ± 122 words) or the EU (2593 ± 140 words), as well as having fewer sentences (*P* < 0.0001), fewer words per sentence (*P* < 0.0001) and fewer syllables per word.

## Discussion

With an increasingly complex burden of care in CF, patients are provided with increasingly large amounts of information to read and comprehend. These include time requirements to read and understand PILs that accompany all aspects of their care, including device inserts, antibiotics and more recently, CFTR medicines. This burden of care generates a demand for clear, empathetic plain language communication, which is crucial for their understanding of the treatment. For patients who may struggle with such information, this information needs to be explained fully and supported with appropriate evidence-based guidance. CF multidisciplinary team (MDT) healthcare professionals should aim to communicate to the PwCF such information at a level that is understood and is commensurate with the patients’ level of health literacy. Communicating at a person’s health literacy level is particularly important for CF patients and their carers, as the quality of their life and health is influenced by knowledge of disease management.^22^ As a result, CF patients have become very knowledgeable about their disease, its treatments and ongoing research.^23^ It also forms a crucial part of promoting patient responsibility and health autonomy, whilst enabling informed, shared decision-making—integral to patient-centred care.^24^ For such a chronic illness as CF, where there is a high burden of care, understanding the need for therapies may contribute to more-active involvement and better adherence of treatments.^25^

To our knowledge, this is the first study to conduct an assessment of the readability of PILs of antibiotics employed in the treatment of respiratory infections in PwCF. In this study, we employed quantitative measurement of words, sentences and syllables, as defined by readability formulae, including FRE, FKGL, Gunning fog and SMOG scores. Readability has become a commonly employed tool to help healthcare professionals prepare patient-facing materials and resources, supported by a growing evidence-base of published literature, where currently there are approximately 500 publications cited in PubMed per year, devoted to its study and application within clinical medicine, particularly its value in patient-facing information and material resources. To date, an advanced PubMed search of the title terms ‘readability’ and ‘antibiotic’ does not return any published articles, which demonstrates the novelty and opportunity of the application of such an approach to promote antibiotic usage awareness health literacy amongst patients.

From examination and comparison of the readability and text metrics results of this study, the US patient information exhibited a statistically significantly better readability score, compared with the EU and UK PILs (Figure 2). Few of the PILs for CF antibiotics from the EU and UK met the recommended FKGL, FRE, Gunning fog or SMOG scores, with a minority of EU and UK PILs achieving an FKGL score of 8 or below and an FRE score of greater than 60. This indicates that EU and UK PILs are not considered to be written adequately for the public and are thus too difficult for the general public to read. Oral, IV and nebulized antibiotics were compared (Figure 5), as it is important to know the readability for antibiotics that the CF patient would administer in their own home, particularly including oral, nebulized and dry powder inhaler antibiotics, as such patients would not have the opportunity to clarify any antibiotic-related queries in real time, unlike in an inpatient setting, where antibiotic queries would be addressed in real time by members of the CF MDT, including the CF pharmacist. There was no statistical difference between the readability, as defined by FRE, between oral and IV-administered antibiotics; however, the nebulized antibiotics had better readability, which was statistically significant (*P* = 0.0043 & *P* < 0.0001, respectively).

When the readability of the antibiotic PILs from this study are compared with another PIL relating to infectious diseases, namely meningococcal vaccination, the readability of the vaccine PIL was slightly better, which had a mean FRE score of 58.1 (versus 52.6 for antibiotics) and a mean FKGL score of 7.3 (versus 8.7 for antibiotics).^26^ Similar findings were also observed in a study that has been conducted to assess the readability of online patient education materials for medical therapy, where it was shown that these materials were poorly written and beyond the level at which the general public was able to read.^27^

PILs are essentially designed to provide the patient with important information regarding their medication, to allow patients the choice and enable them to make knowledgeable and responsible decisions with regard to their medications.^28^ A community-based, cross-sectional survey conducted by a recent study showed that more than half of the participants (64.9%) indicated that reviewing the PILs had positively impacted their medication adherence.^29^ Therefore, it is important that PILs are easily accessible by inclusion in community-dispensed medicines, and are easily read, and their value promoted to patients and service users, by the CF MDT members.

A better knowledge of health-related literature, such as PILs, is often facilitated by improved health literacy; this would enable patients to make more informed management decisions for their health conditions, leading to a potentially better health outcome. Statistics have shown low health-literacy rates are present in almost half of the populations in America and Europe.^30^ Therefore, the importance of good quality, well-written PILs is vital for patients to enhance their health literacy and knowledge of the recommended instruction on their medication. However, a study previously stated that only a small percentage of the PILs now given to patients, in general practices, enhance health literacy (<10%), and they are only appropriate for people with higher levels of education.^31^

Previous studies have also indicated that there are potential links between the reduced level of treatment adherence to low health-literacy rates in patients. As part of an essential strategy to improve adherence, patients must be able to read health-related information regarding their condition.^32^ The ability to comprehend this information may be a crucial method in assisting patients to enhance their general adherence behaviours, which could be a fundamental element while managing chronic diseases.^33^

In the current study, the poor readability results associated with the EU and UK PILs correlate with low word count and sentence count, from comparisons of the text metrics (Figure 3a–d). Significant differences were found when the EU and UK PILs were compared with the US PILs’ word and sentence count (*P* < 0.0001). The better readability score recorded in the US patient information correlates greatly with the text metrics. Compared with the EU and UK PILs, the US information had a significantly lower word count, sentence count and words per sentence within the content of the patient information (Figure 3a–d). Fewer words per sentence within a written piece are associated with improved readability. This result is consistent with what has been reported from a recent study, where improved readability was associated with reduced use of complicated words and reduced sentence length.^34^

Table 1 provides several resources and tools that may help support healthcare and CF MDT authors in the writing of patient information with improved readability.

**Table 1.** Help, support and resources for aiding in the writing of patient information with improved readability

Description	Author(s)	Web address
Best practice guidance on PILs	UK government	https://assets.publishing.service.gov.uk/media/5fe086c18fa8f5149718d66a/Best_practice_guidance_on_patient_information_leaflets.pdf
Developing PILs	The Royal College of Obstetricians and Gynaecologists (UK)	https://www.rcog.org.uk/for-the-public/browse-our-patient-information/developing-patient-information-leaflets/
Simply Put: a guide for creating easy-to-understand materials	CDC (USA)	https://www.cdc.gov/healthliteracy/pdf/simply_put.pdf
Online readability tutor and calculator	Readable.com	www.readable.com
Assessing readability of patient education materials: current role in orthopaedics	Badarudeen S, Sabharwal S	https://doi.org/10.1007/s11999-010-1380-y
Improved readability toolkit and checklist	Anderson H, Moore JE, Millar BC	https://doi.org/10.1016/j.jcf.2021.09.009

The study presented has several limitations. Firstly, the PILs collected and analysed were limited to the English language only. Therefore, all non-English patient information sources were excluded from this study. This was due to the online readability tool (*Readable*) employed in this study being best suited for scoring texts using the English alphabet and is not able to assess readability of texts written with alternative characters, such as Arabic, Chinese and Japanese. Furthermore, whilst the PIL constitutes the formal basis of information on antibiotics to the patient, the PIL may not be the sole source of information for patients. Some patients may receive additional and tailored information on their antibiotics from other healthcare professionals, including pharmacists and doctors, which helps augment this existing information. Examples of these may include visual pill-tracking leaflets, morning/noon/night stickers or food/non-food placed on pill containers. Another limitation of readability parameters was that in materials focusing on health information, frequent repetitions of key phrases for more patient emphasis would result in artefactually elevated FRE and FKGL scores. Word count is an integral component of readability measurements. Most of the major readability algorithms, including the FRE and the FKGL, involve total words in the generation of their respective scores (see Table S1). Word count becomes important when it relates to sentence length, so for example, a large word count contained within a single sentence would have poorer readability compared with the same number of words divided into several short sentences. Therefore, within the context of PILs, those antibiotics with more documented side effects would contain more words. Depending on the sentence structure, this may or may not affect the readability score.

Despite such limitations, readability tests still remain an important parameter to assess and improve the quality of PILs for CF and other chronic disease populations, where adherence to medication is low. Patient adherence to medication in other chronic disease states is also poor, where it has been reported that approximately 50% of chronic disease patients are non-adherent to their respective medical therapy.^35,36^ For example, with stroke patients, it has been reported that *circa* one-third of stroke survivors were non-adherent to their medication.^37^

The value of readability has been evidenced in earlier studies that have simplified patient education materials using readability formulae and resulted in improved comprehension.^38^ Currently, the FDA is consulting on the need for a new and novel form of patient information delivery called ‘Patient Medication Information’, as the FDA recognizes the importance of providing written information to patients about their prescription drugs (https://www.fda.gov/drugs/fdas-labeling-resources-human-prescription-drugs/patient-medication-information), which the FDA notes ‘*can help patients use their prescription drug products safely and effectively, which may reduce preventable adverse drug reactions and improve health outcomes*.’ Where antibiotics are concerned, it is important to convey the potential consequence of AMR development due to failure to adhere to and complete the prescribed antibiotic course.

Low readability scores of PILs and low adherence to such medications still remain major barriers in improving health outcomes in CF. Future studies should focus more on the other factors associated with the improvement of PILs, using larger font size and inclusion of infographics, or even changing the design and layout of the current PILs, which will require discussions between the market authorization manufacturer and the medicines regulator. Additionally, the findings of the current study also pose new research questions, including those around health economics, as well as attempting to correlate improved readability of antibiotic information with better compliance and resistance profiles, which merit further investigation.

## Supplementary Material

dlad129_Supplementary_Data

## Supplementary data

Tables S1 and S2 are available as Supplementary data at *JAC-AMR* Online.
