# Supplementary material for: Improving health literacy of antibiotic use in people with cystic fibrosis (CF)—comparison of the readability of patient information leaflets (PILs) from the EU, USA and UK of 23 CF-related antibiotics used in the treatment of CF respiratory infections
Source: JAC Antimicrob Resist. 2023 Dec 1;5(6):dlad129. doi: 10.1093/jacamr/dlad129 (PMC10691746; doi:10.1093/jacamr/dlad129)
Supplement: dlad129_Supplementary_Data [file dlad129_supplementary_data.zip › JACAMR-2023-099 Table S1.pdf]

Readability Scores	Readability formula	Target score for texts aiming at the general public
**Flesch-Kincaid Grade Level (FKGL)**	0.39 (total words/total sentences) + 11.8 (total syllables/total words) − 15.59	A text aimed at the general audience should have a grade level of 8 or lower. At Grade Level 8, 85% of the general population will be able to read and comprehend the text.
**Flesch Reading Ease (FRE)**	206.835 − 1.015 × (total words / total sentences) − 84.6 × (total syllables / total words)	A target score of 60 or above.
**Gunning Fog Index**	0.4 × [(total words / total sentences) + 100 × (complex words / total words)]	Grade level of 8 or below.
**Simple Measure of Gobbledygook (SMOG) Index**	3 + square root √polysyllabic count in 30 sentences	Grade level of 8 or below.

Supplementary Table 1:- Summary table of readability formulae used in this study and their associated target score for the general public.

Readability formulae are taken from Readable.com
