# Supplementary material for: Improving health literacy of antibiotic use in people with cystic fibrosis (CF)—comparison of the readability of patient information leaflets (PILs) from the EU, USA and UK of 23 CF-related antibiotics used in the treatment of CF respiratory infections
Source: JAC Antimicrob Resist. 2023 Dec 1;5(6):dlad129. doi: 10.1093/jacamr/dlad129 (PMC10691746; doi:10.1093/jacamr/dlad129)
Supplement: dlad129_Supplementary_Data [file dlad129_supplementary_data.zip › Table S2.pdf]

Table S2: Student age and equivalent educational stage in Northern Ireland and USA

Age of student (years)	Stage in Northern Ireland educational system[a]	Stage in US educational system[b]
3-4	Preschool Nursery	Preschool
	Primary School	
4-5	Year 1 (Foundation)	Preschool
5-6	Year 2 (Foundation)	Kindergarten
6-7	Year 3 (Key Stage 1)	Grade 1
7-8	Year 4 (Key Stage 1)	Grade 2
8-9	Year 5 (Key Stage 2)	Grade 3
9-10	Year 6 (Key Stage 2)	Grade 4
10-11	Year 7 (Key Stage 2)	Grade 5
	Secondary School	
11-12	Year 8 (Key Stage 3)	Grade 6
12-13	Year 9 (Key Stage 3)	Grade 7
13-14	Year 10 (Key Stage 3)	Grade 8
14-15	Year 11 (Key Stage 4)	Grade 9
15-16	Year 12 (Key Stage 4)	Grade 10
16-17	Year 13 (Key Stage 4)	Grade 11
17-18	Year 14 (Key Stage 4)	Grade 12
	University/College	

[a] Northern Ireland (NI) Direct. School Curriculum. Curriculum Key Stages. Available from https://www.nidirect.gov.uk/articles/school-curriculum#toc-2

[b] The National Center for Educational Statistics. The structure of education in the United States. Available from https://nces.ed.gov/programs/digest/d02/images/figures/1f_A_1.gif
